# Identifying trade-offs between biodiversity conservation and ecosystem services delivery for land-use decisions

**DOI:** 10.1038/s41598-020-64668-z

**Published:** 2020-05-14

**Authors:** Constance Fastré, Hugh P. Possingham, Diederik Strubbe, Erik Matthysen

**Affiliations:** 1 Evolutionary Ecology Group, Department of Biology, University of Antwerp, Universiteitsplein 1, 2610 Wilrijk, Belgium; 2 Centre of Excellence for Environmental Decisions, School of Biological Sciences, The University of Queensland, Queensland, 4072 Australia; 3 The Nature Conservancy, 4245 Fairfax Drive, Arlington, VA 22203 USA; 4 Center for Macroecology Evolution and Climate, Universitetsparken 15, University of Copenhagen, 2100 Copenhagen, Denmark; 5 Terrestrial Ecology Unit (TEREC), Department of Biology, Ghent University, K.L Ledeganckstraat 32, 9000 Gent, Belgium

**Keywords:** Biodiversity, Conservation biology, Ecosystem services

## Abstract

Sustainable land-use management must account for the potential trade-offs between biodiversity conservation, productive land uses and ecosystem services. In this study, we used Marxan with Zones to generate land use plans that optimize conservation, farming and forestry land uses to reach biodiversity targets while minimizing the opportunity cost for local communities in an inhabited but data-poor National Park in the Andes of Bolivia. Based on six alternative land-use plans, we identified the synergies and trade-offs between the biodiversity benefits achieved in the different plans and the delivery of four locally important water-related ecosystem services modeled with the web-based tool AguAAndes. Although we find synergies between the conservation of high altitude *Polylepis* woodlands and their associated avifauna and three of the ecosystem services investigated, soil erosion levels were actually higher in scenarios with higher achieved biodiversity benefits. Our study shows how systematic conservation planning and ecosystem service delivery modelling can be used to solve land-use conflicts and identify trade-offs between biodiversity conservation and ecosystem services in a data-poor tropical area.

## Introduction

As human population and its need for food, water and shelter increases, conflict between land use and biodiversity intensifies, resulting in the worldwide conversion of natural areas to croplands, pastures, plantations and urban areas at the expense of both biodiversity and environment functioning^1,2^. Land use conversion affects biodiversity mainly through modification or loss of habitat, soil degradation, water depletion and overexploitation of native species^3^ and often results in the disruption of the capacity of the ecosystem to provide services such as water balance and regulation, soil stabilization and air quality^1^.

While ecosystems are converted and fragmented ever faster, the amount and quality of remaining habitats is a key factor influencing whether species can persist or not, particularly for species with small ranges which are characterized by narrow niches and clumped distributions^3^. In recent decades, much conservation effort has been invested in designing protected areas to conserve both fragmented habitats and rare and threatened species from further human degradation and exploitation^4^. There is an increasing demand, however, for protected areas to provide additional functions to that of biodiversity conservation, such as supporting the livelihoods of local communities, providing ecosystem services and/or mitigating the effects of climate change^4,5^. This can however result in conflicting demands, and trade-offs between biodiversity conservation and ecosystem service delivery or human livelihood support have been reported in a variety of places and situations^6^. To efficiently plan for biodiversity protection and management, potential trade-offs must therefore be identified and acknowledged^4,6,7^.

Land use zoning, an informed management practice, is widely used by managers and agencies to resolve conflicts between competing demands^8–11^. While numerous tools are available for systematic conservation planning and zoning, the most widely used are Marxan^12^ and its extension, Marxan with Zones (MarZone^9^). As it allows to assign land parcels to different zones instead of generating a binary output (protected/unprotected), MarZone provides the opportunity to evaluate the consequences and trade-offs of alternative land use plans, resulting in better informed decision making^9^. MarZone was originally created to design marine reserves^8,9^ and only few studies to our knowledge have used the algorithm to perform land use zoning in terrestrial settings^10,11,13–15^.

To explore trade-offs between biodiversity conservation, exploitation and ecosystem service delivery within a terrestrial land-use zoning context, we used the Southern Slope of the Tunari National Park in the Bolivian Andes as a study area. The Southern Slope, facing the city of Cochabamba, is a source of land use conflicts due to its unique biodiversity, concentrated in small *Polylepis* forest remnants, and numerous local communities strongly relying on subsistence agriculture and animal husbandry. Biodiversity is further threatened by afforestation with non-native tree species. Additionally, land use conversion in the study area disrupts ecosystem services, such as water delivery, erosion and runoff control, which are important to both local communities and citizens living in the valley below^16^. Under these problematic circumstances, the local authorities have recently commissioned the establishment of an official management plan for the Tunari National Park. The plan, published in 2017^16^, emphasized the importance to focus management strategies on conserving the *Polylepis* forests of the Southern Slope, because of their unique biodiversity and the ecosystem services they deliver. To supplement this existing management plan, effective until 2026, we generated spatially-explicit management recommendations and explored the potential trade-offs among the conservation of both *Polylepis* forest remnants and suitable bird habitat, opportunity costs for local communities and ecosystem services delivery. To this end, we first generated land use zoning solutions with MarZone aiming at the conservation of biodiversity at minimal opportunity cost, and then evaluated how these solutions affect the distribution of four locally important ecosystem services, and how these services may trade-off with the achieved biodiversity targets.

## Methods

### Study region

**Figure 1:**
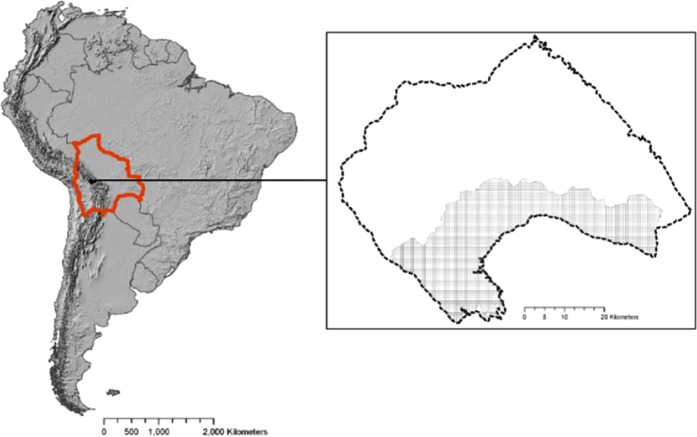
Location of the study area (Southern Slope in light grey divided into 25-ha planning units), within the Tunari National Park (dashed outline in frame) in Bolivia (bold orange outline), South America. Map created using ESRI ArcGIS software version 10.1.

The Southern Slope of the Tunari National Park (TNP, Fig. 1 and Supplementary Fig. S1**)** is a 141,000- ha area ranging from 2750 to 4400 m asl located in the Andes of Bolivia. The TNP was established in 1962 to prevent further degradation of its native vegetation, to conserve its endemic and threatened biodiversity, to protect its delivery of ecosystem services (especially water and erosion control), and to halt the expansion of the city located in the valley^16^. The study region, home to many local communities, was declared as an IBA by BirdLife (IBA BO023^17^) as it supports numerous endemic and/or threatened bird species, among which the endangered Cochabamba Mountain-Finch (*Compsospiza garleppi*) endemic to the mountain ranges of Cochabamba and Potosi provinces^18^. However, the lack of an official management plan of the park and poor enforcement^16^ has resulted in the widespread conversion, especially in the study area, of natural habitats into agricultural fields, pasturelands and exotic tree plantations. It is feared that the forest remnants will disappear while degrading environmental conditions, particularly the additional loss of soil through erosion and the increased water runoff in the area, lead to an increase in natural hazards such as floods and droughts, affecting not only indigenous communities but also the city located downstream^18–21^.

### MarZone framework

Marxan with Zones (referred hereafter as MarZone^9^), uses *biodiversity features* and *economic costs* to allocate land parcels called *planning units* to different land-use categories referred to as *zones*. MarZone is an extension of the decision support tool Marxan^12^ with land use zoning options. MarZone is a complementarity-based algorithm which solves the minimum-set problem using simulated annealing techniques^8,9,22^. Its main task is to find the best zoning plan to meet a set of user-defined biodiversity targets, while minimizing the total cost of the solution.

We divided the study area into 4081 planning units of 25 ha-squares, which reflects a balance between the requirement for analytical time and the resolution of the environmental data available to obtain optimal solutions within reasonable computational time^23^. Planning units were then allocated by the algorithm to different zones representing different possible uses. How much each unit contributes to reach the biodiversity targets depends on its *current biodiversity value* (the percentage suitability of the unit for each of the species and percentage of *Polylepis* cover, see below) and the zone to which it is allocated (through the *zone contribution*, see below). Each planning unit receives a cost for allocating the unit to one of the zones. Note that the analysis does not consider possibilities for habitat restoration; therefore, the current biodiversity value of a planning unit cannot be increased.

### Biodiversity features and targets

We used two categories of biodiversity features in the analysis: one habitat feature (actual *Polylepis* woodland cover), and the habitat suitability for 35 bird species (Table 1) occurring in *Polylepis* woodlands in our study area (*i.e*. species for which shrubland and forest habitats are considered either major or suitable to the species according to BirdLife International^24^ and/or recorded regularly within the surveyed *Polylepis* woodlands, see extended methods S1). Of these species, three are considered Polylepis-dependent species as they rely on the tree species to feed and five species are considered *Polylepis*-associated species as they occur in *Polylepis* habitats throughout their range^25^ (Table 1). We calculated the *Polylepis* cover as the percentage of current *Polylepis* forest cover per planning unit, estimated from a Landsat 8 image (30*30 m resolution, see extended methods S2 for details). Habitat suitability models for all 35 bird species were generated from occurrence data collected in the field (see extended methods S1 for details) using the Maxent methodology (version 3.1; http://www.cs.princeton.edu/~schapire/maxent/^26^. To prevent the selection of unsuitable habitat by MarZone, we thresholded each species’ continuous habitat suitability estimates using the True Skill Statistics (TSS^27^). For each species, values located below the threshold calculated (Supplementary Table S1), were converted into 0 values while values above the threshold remained unchanged to reflect ‘true’ suitability^28^. The habitat suitability value per planning unit was then calculated from the thresholded maps, as the mean suitability per planning unit for each of the bird species.

We calculated the target of each biodiversity feature based on its conservation priority^29^. To test how targets affected land-use zoning, we ran three different scenarios with different target ranges for biodiversity features depending on their conservation priority. We set arbitrary minimum and maximum targets as 20% and either 50%, 75% or 90% of the total amount of each biodiversity feature with lowest and highest conservation priority, respectively. For features with intermediate conservation priority scores, we applied a linear scale to the total species scores from a minimum of 20% up to either 50%, 75% or 90% (Table 1). Conservation priority was defined as follows: for each bird species, a score of 1 was given for country endemism, IBA trigger species, EBA trigger species and small range size (<75000 km², BirdLife International^24^). Additionally, a score of 1 and 2 were given, respectively, to species classified as Near-Threatened or Endangered by IUCN^30^. We systematically assigned the maximum target for *Polylepis* cover (*i.e*. 50%, 75% or 90% of current total cover distribution, depending on the scenario).

**Table 1:** Biodiversity features with their rarity/endemism scores (Total score), their corresponding target values for the three target ranges (Target50 = 20–50; Target75 = 20–75 and Target 90 = 20–90) and the contribution values to the agroforestry (AFO), conservation and bare zones (CON and BARE) and forestry and grazing zones (FOR and GR).

Biodiversity features	Total score	Target values per scenario			Contribution value to zones			
		50%	75%	90%	AFO	AGR	CON/BARE	FOR/GR
**Habitat feature**								
*Polylepis* cover	7	50	75	90	0.2	0	1	0
**Bird species (habitat suitability)**								
*Agelaoides badius*	0	20	20	20	0.8	0.5	1	0
*Ampelion rubrocristatus*	0	20	20	20	0.2	0	1	0
*Anairetes flavirostris*	0	20	20	20	0.4	0	1	0
*Anairetes parulus*	0	20	20	20	0.2	0	1	0
Asthenes dorbygnyi*	0	20	20	20	0.6	0	1	0
*Asthenes heterura*	5	41	59	70	0.4	0	1	0
*Atlapetes fulviceps*	1	24	28	30	0.4	0	1	0
*Catamenia inornata*	0	20	20	20	1	1	1	0
*Cinclodes atacamensis*	0	20	20	20	0.8	0.5	1	0
*Cinclodes fuscus*	0	20	20	20	0.8	0.5	1	0
*Colaptes rupicola*	1	24	28	30	0.6	0	1	0
Compsospiza garleppi*	7	50	75	90	0.6	0	1	0
Conirostrum binghami**	3	33	44	50	0.2	0	1	0
*Conirostrum cinereum*	0	20	20	20	0.4	0	1	0
*Diglossa carbonaria*	4	37	51	60	0.6	0	1	0
*Lepasthenura fuliginiceps*	0	20	20	20	0.4	0	1	0
*Melanopareia maximilianus*	0	20	20	20	0.6	0	1	0
*Myioborus bruniceps*	0	20	20	20	0.4	0	1	0
Ochthoeca leucophrys*	0	20	20	20	0.4	0	1	0
*Ochthoeca oenanthoides*	1	24	28	30	0.2	0	1	0
*Oreopsar bolivianus*	4	37	51	60	0.8	0.5	1	0
*Patagona gigas*	0	20	20	20	0.4	0	1	0
Phacellodomus striaticeps*	1	24	28	30	0.2	0	1	0
*Phrygilus atriceps*	0	20	20	20	0.2	0	1	0
*Poospiza hypochondria*	0	20	20	20	0.6	0	1	0
Pseudosaltator rufiventris*	5	41	59	70	0.6	0	1	0
*Psilopsiagon aymara*	0	20	20	20	0.4	0	1	0
*Saltator aurantiirostris*	0	20	20	20	0.8	0.5	1	0
*Sappho sparganura*	0	20	20	20	0.4	0	1	0
*Scytalopus simonsi*	2	29	36	40	0.4	0	1	0
*Sicalis olivascens*	0	20	20	20	0.8	0.5	1	0
Spinus crassirostris**	1	24	28	30	0.2	0	1	0
Sylviorthorhynchus yanacensis**	4	37	51	60	0.2	0	1	0
*Troglodytes aedon*	0	20	20	20	0.6	0	1	0
*Zonotrichia capensis*	0	20	20	20	1	1	1	0

*Indicate Polylepis-associated and ** indicate Polylepis-dependent species.

We built six scenarios with the three target ranges, applied to scenarios with either four or five zones (Table 1), which we named ‘4z50’, ‘4z75’, ‘4z90’, ‘5z50’, ‘5z75’ and ‘5z90’. For each scenario, we used the best solution (*zoning plan*) found by MarZone from 100 iterations and calculated its *achieved biodiversity value*. The achieved biodiversity value of each zoning plan is calculated as the average of all habitat suitability and *Polylepis* forest cover percentages conserved in the zoning plan across planning units. This achieved biodiversity value was later plotted against the estimates of ecosystem service delivery to identify synergies and trade-offs (see below).

### Land use allocation rules

We identified five different land use zones to which planning units could be allocated by MarZone. Three of the land-use zones were based on the most common extractive land use types carried out in the study area: *agriculture, grazing* and *forestry zones*. The agriculture zone includes intensive, extensive or subsistence agricultural practices primarily aimed to grow potatoes or cereals (see Extended Methods S3 for details). In practice, the type of agricultural practices carried out in the zone is constrained by altitude, slope and water availability^19^. In the grazing zone, animal husbandry with sheep or cows is practiced, while forestry refers to the establishment of dense exotic tree plantations. We included a fourth zone, the *conservation zone*, which represents a strictly protected strategy, with no further human exploitation allowed. Since it has been suggested in previous local studies that agroforestry practices (fields planted with native trees such as *Polylepis*) would be beneficial both for biodiversity and ecosystem service delivery^17,31,32^, we ran an additional set of scenarios in which we included a fifth *agroforestry zone*. Finally, 6% of the study area, mainly covered by rocks and unsuitable for anthropogenic use, were locked into a static *bare zone* and retain any biodiversity value they may have.

To ensure land use allocation remains realistic, we established land use allocation rules for each planning unit based on the current land cover (derived from the classified Landsat image mentioned above, Supplementary Fig. S4) and the best potential land use (Supplementary Fig. S5). The best potential land use was extracted from a map previously published^19^ based on local expert knowledge and data on topography, vegetation cover and soil characteristics. We determined the best potential land use for each planning unit as the most dominant best potential land use within that unit. As the best potential land use is constrained by the properties or capacity of the land (*i.e*. only high-quality land can be used for agriculture while steep areas or poor soils can only be used for grazing and forestry purposes), we established four allocation rules: (1) all planning units can be allocated to the conservation zone; (2) planning units with agriculture as best potential land use can be allocated to any zone; (3) planning units can be allocated to forestry and grazing if their best potential use is either forestry or grazing; (4) any planning unit can be allocated to agriculture or grazing if it is currently covered by puna grassland/agricultural fields and to forestry if it currently contains exotic plantations.

### Contribution of each land use to conservation

We used the zone contribution feature of MarZone to specify how the value of biodiversity features of each unit contributes to the targets when allocated to a specific zone. As planning units allocated to the conservation zone or to the bare zone are assumed to be unaffected by land use, their biodiversity values are also unaffected, and we assigned a 100% contribution value to both zones for all features. Both forestry and grazing zones were assigned a 0% contribution for all features, as forestry (which is exclusively carried out with exotic tree species) and grazing practices strongly impact and may ultimately displace native vegetation and its avifauna^31,33–35^. For agriculture and agroforestry, zone contributions were differentiated among bird species based on our own detailed observations on bird habitat use in the *Polylepis-*agriculture mosaic habitats characteristic of the study area (Fastré *et al*., in prep) in combination with literature data^36–38^ and BirdLife habitat classifications^24^. Combining data from these different sources, we classified the species by their habitat affinity into three categories: species observed (1) exclusively in forested habitats (native forests), (2) in both forested and agricultural habitats and (3) mainly in agricultural habitats. We arbitrarily assigned contribution values of 0, 50 and 100% to the agricultural zone for bird species in each respective category. We further subdivided species observed in both forests and agricultural habitats into species with (2a) higher affinity for forest, (2b) equal affinity for both habitats and (2c) higher affinity for agriculture. We assigned contribution values of 100, 80, 60, 40 and 20% to the agroforestry zone for bird species belonging to categories (1), (2a), (2b), (2c) and (3), respectively (Table 1).

### Costs of planning units

We estimated the opportunity cost of planning units as the loss of potential benefit to landowners of practicing a specific land use instead of the best potential land use. For this we converted the best possible land use map^19^ to a one-hectare resolution raster, using the most common land use as new land use per one-hectare pixel. From the same study, we derived values (in USD per hectare) for the maximum potential benefit of the land uses corresponding to the different zones (Table 2). We arbitrarily estimated that the potential monetary benefit of agroforestry, for which we did not have any value, would be 80% of the corresponding agricultural land use. We calculated the opportunity cost per hectare as the difference between the maximum potential benefit of the best possible land use, and the land use potentially allocated by MarZone (Table 2). We summed costs per hectare within each 25-hectare planning unit to obtain the values used in the analysis.

**Table 2:** Maximum potential benefit of the best potential land use type per hectare (USD) and opportunity cost for each allocated land use zone per hectare (USD). Type I agriculture and grazing uses are carried out on soil that are more productive than Type II uses.

Best Potential Land Use	Max Potential Benefit (USD)	Opportunity Cost Per Zone (USD)				
		Agriculture	Grazing	Forestry	Conservation	Agroforestry
Agriculture - Type I	2075	0	2065	1190	2075	415
Agriculture - Type II	1525	0	1515	640	1525	305
Grazing - Type I	10	0	0	0	10	2
Grazing - Type II	5	0	0	0	5	1
Forestry	885	0	875	0	885	177
Conservation	0	0	0	0	0	0

### Ecosystem services modeling

We used the web-based tool AguAAndes (http://www.policysupport.org/aguaandes), a process-based hydrological model which incorporates detailed spatial datasets at one square kilometer and one hectare resolution for the Andes and scenarios for biophysical and socioeconomic processes and climate, land use and economic change to support hydrological analysis and decision-making in these data-poor areas^39–43^. We modeled four water-related ecosystem services that have high relevance for the study region^19^: (1) erosion leading to agricultural capacity loss, estimated by net soil thickness loss (mm/year); (2) flood risk in areas beneath the slope caused by water runoff, estimated by total surface water runoff (m³/year); (3) water stress, estimated as the percentage of non-agricultural unmet water demand which can reflect long-term underground water reserves depletion; and (4) water pollution estimated as the percentage of water potentially contaminated by human activity.

We developed a baseline model for each service (Supplementary Figs. S6–S9), representing the current situation, which was calculated using globally available and remotely sensed data. The hydrological baseline is simulated as a mean over the period 1950 and 2000^39^. Baseline total values of erosion (mm/yr), runoff (m³/yr), water stress (summed %) and water pollution (summed %) were calculated and compared with the values calculated for the land use plans generated with MarZone (see below). This modeled baseline situation represents ecosystem services in a study area that would be entirely conserved in its current state. We then used simulations to evaluate the effect on each ecosystem service of converting the entire study area to each of the zone used in the zoning plan. To assess the effects of (agro)forestry and grazing, we simulated ecosystem services under afforestation and conversion to grasslands, respectively. To assess the effects of conversion to agriculture we simulated deforestation to model erosion and runoff, because cultivated fields remain devoid of any vegetation cover most of the year. However, since crop cover and agricultural practices such as irrigation may have strong effects on water stress and pollution, we modeled these two services under simulated conversion to croplands.

To obtain the values of ecosystem services for the different zoning plans obtained previously, we overlaid the planning units allocated to each zone in each of the MarZone solution with the corresponding ecosystem service simulation (afforestation for forestry and agroforestry, deforestation or crop conversion for agriculture, grassland conversion for grazing and baseline situation for the conservation and bare zones). We summed ecosystem service values of the different zones to estimate the total values of erosion (mm/yr), runoff (m³/yr), water stress (summed %) and water pollution (summed %) for each zoning plan generated with MarZone. We also calculated the change in delivery of these ecosystem services compared to the current situation as the difference between the baseline situation and the new values per zone and for the entire study area. Finally, to identify potential trade-offs, the summed ecosystem service values of each zoning plan were plotted against the average achieved biodiversity value.

## Results

### Zoning plans

**Figure 2:**
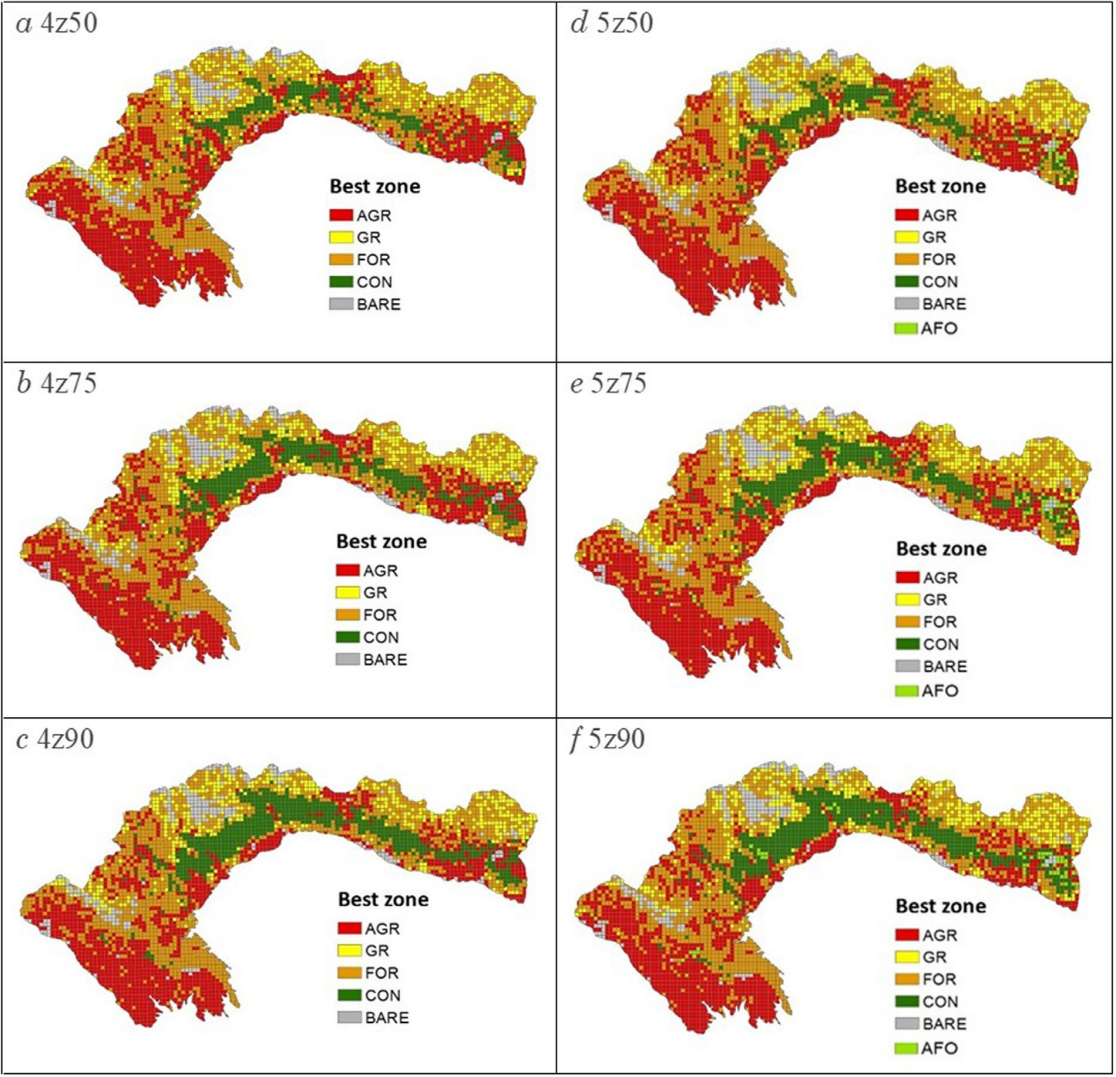
Zoning plans for the six scenarios (AGR = Agriculture, GR = Grazing, FOR = Forestry, AFO = Agroforestry and CON = Conservation). On the left column are the zoning plans for scenarios with 4 zones and (**a**) 50%, (**b**) 75% and (**c**) 90% maximum targets. On the right column are the zoning plans for the corresponding scenarios with 5 zones.

The zoning plans (Fig. 2) reached all their biodiversity targets for each of the six scenarios. Planning units allocated to the conservation zone are mainly localized in the center and eastern mid-elevation area of the study area, forming one clumped area that expands with increasing targets (Fig. 2), at the expense of units allocated to forestry and to a lesser extent agriculture and grazing (Supplementary Fig. S2). For the lowest biodiversity targets (4z50 and 5z50 scenarios), the best solutions achieved all targets when 8% of the total area is set aside for conservation. This increases to a little more than 11% and 15% of the total area at intermediate and highest biodiversity targets respectively. As MarZone only assigned one to two percent of the planning units to agroforestry, land use allocation is rather similar in scenarios with 4 and 5 zones (Fig. 2 and Supplementary Fig. S2). The cost of the solutions increases with targets but is slightly lower when including agroforestry as a land use option (Supplementary Fig. S3).

### Ecosystem services and land use planning

**Figure 3:**
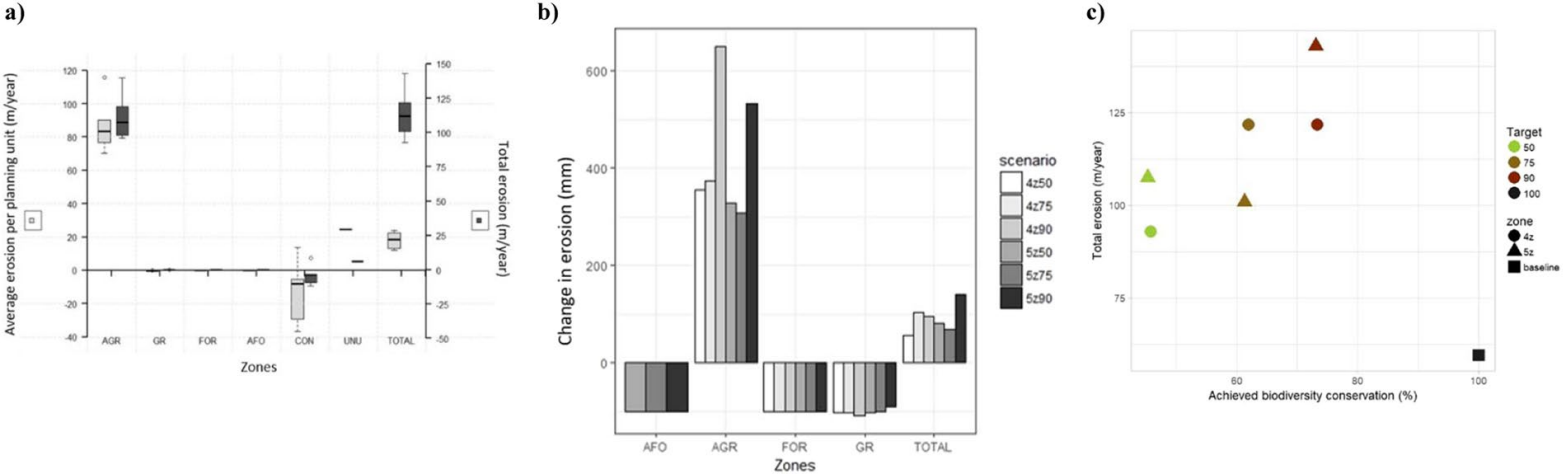
Comparison of erosion values across scenarios and zones and trade-off between erosion and achieved biodiversity value. (**a)** Average erosion per planning unit (left axis) and total erosion values (right axis) in mm/year, for each zone (AGR = agriculture, GR = grazing, FOR = forestry, AFO = agroforestry, CON = conservation and BARE) and for the entire area (TOTAL) for the zoning plans of six alternative scenarios generated by MarZone. (**b)** Difference in erosion (mm) across zones and in the study area compared to the baseline situation. Scenarios refer to the number of zones (4 or 5) and biodiversity targets (50, 75, 90%). (**c)** Achieved biodiversity value (average conserved percentage across biodiversity features), plotted against erosion values (m/year) for each zoning plan generated for the six MarZone scenarios and the current situation (baseline).

There is an estimated total net erosion (erosion minus deposition) of 106 meters of soil thickness per annum in the study area (18 mm per planning unit) on average across the scenarios’ zoning plans. This corresponds to an increase of 91.1% (or 54.2 meters) on average compared to the current situation (Fig. 3a,b). Most erosion occurs in the planning units converted to agriculture, where levels increase by 424.7% on average, influencing both total and average erosion values for the entire study area. Conversely, there is a decrease in erosion in planning units of the forestry and agroforestry zones and to a lesser extent in the grazing zone, thanks to the stabilizing effect of forest and grass cover. Interestingly, planning units of the conservation zone show negative erosion values or net average soil deposition (Fig. 3a). Peaks in the increase of erosion in the agriculture zone at higher targets suggest that this zone includes units very sensitive to erosion (located on steeper slopes or less vegetated), which are therefore particularly strongly affected by agricultural activities (Fig. 3b). As shown on Fig. 3c, we find a strong trade-off between biodiversity conservation and erosion control across the six scenarios, as higher achieved biodiversity values are associated with zoning plans with increased erosion.

**Figure 4:**
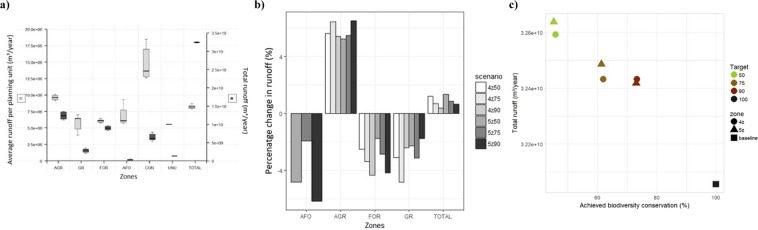
Comparison of runoff values across scenarios and zones and trade-off between erosion and achieved biodiversity value. (**a)** Average runoff per planning unit (left axis) and total runoff values (right axis) in m³/year, for each zone (AGR = agriculture, GR = grazing, FOR = forestry, AFO = agroforestry, CON = conservation and BARE) and for the entire area (TOTAL) for the zoning plans of six alternative scenarios generated by MarZone. (**b)** Percentage change in runoff (%) across zones and in the study area compared to the baseline situation. Scenarios refer to the number of zones (4 or 5) and biodiversity targets (50, 75, 90%). (**c)** Achieved biodiversity value (average conserved percentage across biodiversity features), plotted against runoff values (m³/year) for each zoning plan generated for the six MarZone scenarios and the current situation (baseline).

We find an average of 8.2 million m³/year water runoff per planning unit across the six zoning plans. Although runoff levels in the study area are higher in all the solutions than the current level (+0.9% on average), differences become smaller as biodiversity targets increase (Fig. 4a,b). Highest average water runoff values per planning unit are found in the conservation zone, followed by units allocated to agriculture. The latter experience an increase in runoff compared to the current situation (+5.8% on average across scenarios). Conversely, planning units converted to forestry and agroforestry exhibit a decrease in runoff (−3.2% and −4.3% on average across all scenarios respectively, Fig. 4b). There is no trade-off between biodiversity conservation and runoff control as total runoff decreases with achieved biodiversity values (Fig. 4c).

**Figure 5:**
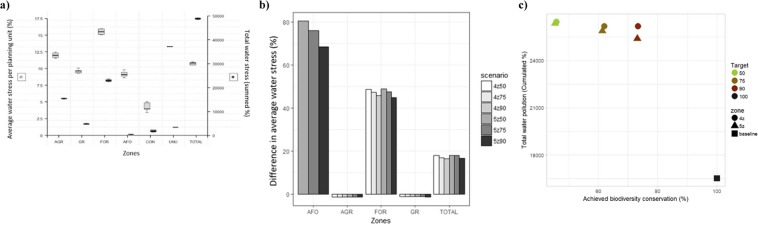
Comparison of water stress values across scenarios and zones and trade-off between erosion and achieved biodiversity value. (**a)** Average water stress per planning unit (%, left axis) and total water stress values (summed percentage, right axis), for each zone (AGR = agriculture, GR = grazing, FOR = forestry, AFO = agroforestry, CON = conservation and BARE) and for the entire area (TOTAL) for the zoning plans of six alternative scenarios generated by MarZone. (**b)** Difference in average water stress (%) across zones and in the study area compared to the baseline situation. Scenarios refer to the number of zones (4 or 5) and biodiversity targets (50, 75, 90%). (**c)** Achieved biodiversity value (average conserved percentage across biodiversity features), plotted against total water stress values (summed %) for each zoning plan generated for the six MarZone scenarios and the current situation (baseline).

Water stress increases by 17.4% on average across the different zoning plans, reaching average values of 10.8% per planning unit (Fig. 5a,b). Highest levels of water stress are found in units converted to forestry and agriculture (16 and 12% on average). The strongest increase in water stress compared to current levels occur in both agroforestry and forestry zones (+75.0% and +47.2% on average), caused by the transformation of large amounts of water by trees into evapotranspiration. Despite a marginal decrease in water stress (−1.2%), units placed in the agriculture zone exhibit high average stress values (12%) compared to units in the grazing and conservation zones (9.6 and 4.2% respectively). Planning units of the conservation zone have the lowest mean water stress values of any zone. Figure 5c shows again no trade-off between biodiversity conservation and water stress, as the total amount of unmet water demand across the study area (summed percentages) slightly decreases with achieved biodiversity values.

**Figure 6:**
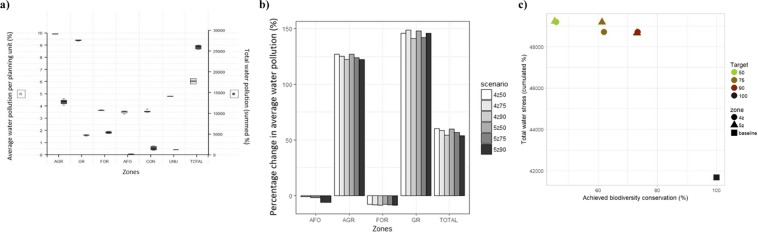
Comparison of water pollution values across scenarios and zones and trade-off between erosion and achieved biodiversity value. (**a)** Average water pollution per planning unit (%, left axis) and total water pollution values (summed percentage, right axis), for each zone (AGR = agriculture, GR = grazing, FOR = forestry, AFO = agroforestry, CON = conservation and BARE) and for the entire area (TOTAL) for the zoning plans of six alternative scenarios generated by MarZone. (**b)** Percentage change in average water pollution (%) across zones and in the study area compared to the baseline situation. Scenarios refer to the number of zones (4 or 5) and biodiversity targets (50, 75, 90%). (**c)** Achieved biodiversity value (average conserved percentage across biodiversity features), plotted against total water pollution values (summed %) for each zoning plan generated for the six MarZone scenarios and the current situation (baseline).

Water pollution increases by an average of 57.2%, reaching values of 6% (Fig. 6a,b). This overall increase is caused by the high water pollution levels in units converted to agricultural and grazing zones, with 2 to 3-fold increases compared to the baseline situation. Afforested units of the forestry and agroforestry zone slightly mitigate water pollution, decreasing baseline pollution levels by on average 8.3% and 3.1% across solutions, respectively. Despite similar total water pollution values across scenarios, there is a decrease in the summed pollution percentages with increasing biodiversity targets, and water stress is higher when considering agroforestry as a potential land use in the area (i.e. in the 5-zones scenario). As targets and the number of zones increase, fewer planning units are allocated to agricultural land use as the conservation and agroforestry zones expand, resulting in less water being polluted overall. There is therefore no trade-off between biodiversity conservation and water pollution control (Fig. 6c).

## Discussion

Our study shows how systematic conservation planning, in conjunction with online policy support tools such as AguAAndes, can be used to provide information on land use conflicts in data-poor but biodiverse protected areas. Using Marxan with Zones to find optimal land use zoning plans, we identify trade-offs between biodiversity conservation and ecosystem service delivery (Figs. 3–6**)** to inform conservation management in the Tunari National Park. Our analysis shows it is possible to reach all biodiversity conservation targets in the study area, including the protection of 90% of the current *Polylepis* cover and 90% of the suitable habitat of high-priority conservation species such as the threatened Cochabamba mountain finch (*C. garleppi*). Reaching these targets requires only 8 to 15% of the area to be set aside for conservation, thus leaving 85% available to other land uses such as agriculture and forestry.

We find a synergy between the land use plans achieving higher biodiversity benefits and three of four locally important water-related ecosystem services. However, increasing biodiversity benefits comes at the expense of soil stabilization across the study area. Indeed, while runoff, water stress and water pollution levels decrease linearly with achieved biodiversity values, we observe a strong increase in erosion with achieved conservation values across the six scenarios. Even though this increase in erosion may seem counter-intuitive as more units are conserved, higher erosion results from the conversion of erosion-prone units to agriculture so that biodiverse units are conserved. One way of avoiding such an undesirable outcome would be to prevent agricultural activities in areas located on steep slopes^16^. Alternatively, to mitigate erosion and avoid the loss of steeper areas to production, we advocate for the use of agroforestry instead of agriculture in these erosion-prone areas. Even though agroforestry did not perform better than strict conservation to balance biodiversity benefits and opportunity costs in our zoning plans, our models show that it can mitigate erosion, runoff and pollution. We therefore suggest that the potential role of agroforestry in helping to conciliate biodiversity and ecosystem service delivery goals in the study area and other similar montane environments should be further explored.

In our study area, agricultural activities have the most pronounced negative effects on ecosystem service delivery, and these effects are often stronger than the mitigating influence of (agro-)forestry activities. Our finding that more biodiversity protection goes hand in hand with increased ecosystem service delivery is probably due to the fact that protecting biodiversity means committing more land to a strict conservation, no human-use strategy, instead of allowing agriculture activities. However, even if an overall increase in ecosystem service delivery associated with increasing biodiversity benefits means that there is an improvement in runoff mitigation and water provision and quality on the Tunari National Park’s Southern Slope, and by extension in the city located downstream, local effects in each zone should also be considered as they can affect local livelihoods. The combination of increased erosion and runoff levels in planning units converted to agriculture, for instance, may mean diminished agricultural capacity and productivity in the future, forcing locals to turn to new agricultural land^1^. Additionally, with higher amounts of water available on the ground surface of agricultural land (decreased water stress), the high levels of erosion and runoff may equate to increased risks of floods downstream.

The most biodiverse planning units, placed in the conservation zone, exhibit particularly low levels of erosion and water stress and the highest amounts of runoff. Such attributes could be explained by a combination of high native vegetation cover and high precipitation levels. Protection of biodiverse areas is thus particularly important as vegetation clearance resulting from land use conversion, in combination with the high levels of precipitation, could be expected to cause more erosion and runoff-related problems than elsewhere in the study area. Even though forest conservation often constitutes a trade-off when considering water quantity regulation, due to high intake and evapotranspiration of trees^40^, *Polylepis* woodlands appear to be an exception, as conserved planning units exhibit very low water stress values. Indeed, in addition to these units receiving higher amounts of rain, native high-elevation trees such as *Polylepis* trees, through their high leaf-area indices, can locally increase water balance by capturing more rainwater than exotic, planted trees^32^.

While the ecosystem services models created with AguAAndes provide useful information as to how land-use patterns in the study area can comparatively affect runoff, erosion, water balance and water pollution in our study area, we uncovered a few limitations in the model. First, we suspect that the effects of grazing on both runoff and erosion are underestimated by the model. It has been shown that trampling and grassland burning, commonly carried out to promote shoot regeneration necessary for sheep and cattle to forage in the High Andes, strongly deteriorate soils, causing high levels of erosion and soil compaction and decreasing their water retention capacities^32,44,45^. It is generally thought that native camelid grazers such as alpacas and llamas, thanks to their soft feet and ability to feed on dry grasses, do not affect the environment as strongly as introduced livestock^19,32,46^. Second, afforestation simulations do not consider differences among tree species and their potential adaptations to the environment. Previous studies reported important differences in soil stabilization, water retention and transpiration properties between introduced exotics (*Pinus* and *Eucalyptus* sp.) and native trees in many areas in the Andes^34,47–49^. Because the afforestation simulations were built using global datasets, we argue they are sufficiently accurate to evaluate the relative effects of exotic forestry activities on ecosystem services delivery. However, ecosystem service delivery in the agroforestry zone might differ if native trees are planted to benefit biodiversity. Third, because of a lack of data, factors such as agriculture intensity, fertilizer or pesticide use could not be included in the calculation of water quality, although they may have critical effects. While such ecosystem services models are unavoidably associated with uncertainty, they are useful to compensate for the lack of empirical data available^50,51^. To deal with this uncertainty, we suggest making flexible management recommendations that should be adapted when empirical data on ecosystem services distribution patterns becomes available^50,51^.

Our analysis shows that in conflicted areas such as the Southern Slope of the TNP, the use of conservation planning and modeling tools can uncover ways to conciliate production land uses and biodiversity conservation on one hand and identify trade-offs and synergies between biodiversity conservation and other ecosystem services on the other hand. We found that in our study area, biodiversity conservation can also benefit ecosystem service delivery and thus increase human well-being. However, large scale conversion to agricultural land-use, even if it can improve local livelihoods, would have negative effects on the environment through large increases in erosion and runoff values in converted areas, and an increased risk of floods downstream. More sensitive agricultural practices or the use of native trees to stabilize soils could however alleviate these problems^18,52^. While exotic forestry activities provide multiple benefits to people such as erosion, runoff and water pollution mitigation, they may be problematic in some areas due to their large water intake. Such plantations do not however provide any benefit for biodiversity and may replace the native vegetation when they are installed too close to *Polylepis* patches^34^. Finally, as both rainfall and temperature patterns are expected to be strongly disrupted by climate change in high-altitudes ecosystems^37,53,54^, potentially affecting water-related ecosystem services, the need for efficient protection of the remaining biodiverse areas in our study area is urgent. Restoration of existing *Polylepis* remnants likely is a first useful step towards better water regulation and provision, mitigating runoff and erosion, as well as contributing to biodiversity conservation in the face of an uncertain future. However, to better protect biodiversity and ecosystem services delivery in our study area, further research is needed to generate and integrate more empirical data on (1) the distribution and management needs of species belonging to other taxa and/or associated with other ecosystems present in the TNP such as *puna* grasslands, other native low-elevation forests or Andean lakes and bogs^16^ and (2) on the spatial distribution and the synergies and tradeoffs between biodiversity conservation and ecosystem services delivery for the ecosystem services considered in our analysis as well as for other important ecosystem services (e.g. pollination).

The land use plans generated in this study constitute a useful spatially-explicit basis to supplement the recommendations from the official management plan^16^ and support decision making by local managers in the study area. These plans are however not designed to be implemented as they are and without further consideration, but should be considered as initial suggestions that will require further interaction with and engagement from stakeholders^13,14^, in particular the local communities inhabiting the area^55^. Even though the land plans we have generated are designed to minimize opportunity costs in the study area, their implementation would likely affect some local communities more than others because different land uses have different implementation and management costs^13,14^. It is therefore crucial, before considering stricter enforcement of regulations in the TNP, to find ways to balance the immediate local needs and the long-term protection of natural habitats and ensure local communities can benefit from conservation management strategies, for example through community-based reforestation and conservation initiatives^52,55–57^.

## Supplementary information

Supplementary information.

## Data Availability

The datasets generated during and/or analysed during the current study that are not included in this published article and its Supplementary Information files are available from the corresponding author on reasonable request.

## References

[1] Foley JA (2005). Global consequences of land use. Science (80-.)..

[2] Gibson L (2011). Primary forests are irreplaceable for sustaining tropical biodiversity. Nature.

[3] Pimm SL, Raven P (2000). Biodiversity: Extinction by numbers. Nature.

[4] Watson JEM, Dudley N, Segan DB, Hockings M (2014). The performance and potential of protected areas. Nature.

[5] Naughton-Treves L, Holland MB, Brandon K (2005). The Role of Protected Areas in Conserving Biodiversity and Sustaining Local Livelihoods. Annu. Rev. Environ. Resour..

[6] McShane TO (2011). Hard choices: Making trade-offs between biodiversity conservation and human well-being. Biol. Conserv..

[7] Bruner AG, Gullison RE, Balmford A (2004). Financial Costs and Shortfalls of Managing and Expanding Protected-Area Systems in Developing Countries. Bioscience.

[8] Parker SR, Truscott J, Harpur C, Murphy SD (2015). Exploring a Approach to Spatial Planning in Fathom Five National Marine Park, Lake Huron, Canada, using Marxan with Zones. Nat. Areas J..

[9] Watts ME (2009). Marxan with Zones: Software for optimal conservation based land- and sea-use zoning. Environ. Model. Softw..

[10] Law, E. A. *et al*. Mixed policies give more options in multifunctional tropical forest landscapes. *J. Appl. Ecol*., 10.1111/1365-2664.12666 (2016).

[11] Adams VM, Pressey RL, Álvarez-Romero JG (2016). Using optimal land-use scenarios to assess trade-offs between conservation, development, and social values. PLoS One.

[12] Ball, I. R., Possingham, H. P. & Watts, M. E. Marxan and relatives: software for spatial conservation prioritisation. in *Spatial conservation prioritisation: Quantitative methods and computational tools* 185–195 (2009).

[13] Wilson AKA (2010). Conserving biodiversity in production landscapes. Ecol. Appl..

[14] Reyers B, O’Farrell PJ, Nel JL, Wilson K (2012). Expanding the conservation toolbox: Conservation planning of multifunctional landscapes. Landscape ecol..

[15] Mehri A, Salmanmahiny A, Momeni Dehaghi I (2017). Incorporating zoning and socioeconomic costs in planning for bird conservation. J. Nat. Conserv..

[16] SERNAP-FAUNAGUA. *Plan de Manejo del Parque Nacional Tunari*. (2017).

[17] BirdLife International. Important Bird Areas factsheet: Southern slopes of Tunari National Park (Vertiente Sur del Parque Nacional Tunari IBA). (2017). Available at, http://www.birdlife.org. (Accessed: 11th May 2017).

[18] Balderrama JAD (2006). endemism and conservation issues of the avifauna of Tunari National Park (Cochabamba, Bolivia). Ecolog?a en Bolivia.

[19] Sanabria Siles, N., Auza Aramayo, M., Dalence Martinic, J., Herrera, B. & Avilés Ribera, S. *Aptitud de aprovechamiento sostenible y de conservación del parque nacional Tunari*. (2012).

[20] FAUNAGUA. *Plan de manejo Parque Nacional Tunari - Caracterizacion integral de la fauna y propuesta de zonificacion específica*. (2015).

[21] SERNAP. Parque Nacional Tunari: Información general. Available at, http://www.sernap.gob.bo/index.php?option=com_content&view=article&id=82&Itemid=285. (Accessed: 6th June 2016) (2016).

[22] Sarkar S (2006). Biodiversity Conservation Planning Tools: Present Status and Challenges for the Future. Annu. Rev. Environ. Resour..

[23] Ardron, J. A., Possingham, H. P. & Klein, C. J. *Marxan Good Practices Handbook, Version 2*. *Pacific Marine Analysis and Research Association* (2010).

[24] BirdLife Data Zone. BirdLife International, Species factsheet Available at, http://www.birdlife.org/datazone. (2016).

[25] Fjeldsa, J., Krabbe, N. Birds of the High Andes. 1990.

[26] Phillips, S. J., Anderson, R. P., Schapire, R. E. Maximum entropy modeling of species geographic distributions. *Ecological Modelling* 190 (3–4):231–259 (2006).

[27] Allouche O, Tsoar A, Kadmon R (2006). Assessing the accuracy of species distribution models: Prevalence, kappa and the true skill statistic (TSS). J. Appl. Ecol..

[28] Wilson KA, Westphal MI, Possingham HP, Elith J (2005). Sensitivity of conservation planning to different approaches to using predicted species distribution data. Biol. Conserv..

[29] Lessmann J, Muñoz J, Bonaccorso E (2014). Maximizing species conservation in continental Ecuador: A case of systematic conservation planning for biodiverse regions. Ecol. Evol..

[30] IUCN. The IUCN Red List of Threatened Species. Available at, http://www.iucnredlist.org. (Accessed: 25th October 2018) (2018).

[31] Hjarsen, T. Biological diversity in high altitude woodlands and plantations in the bolivian Andes: implications for development of sustainable land-use. in *III Simposio Internacional de Desarollo Sustentable de Montañas: entiendo las interfaces ecológicas para la gestión de los paisajes culturales en los Andes* 145–149 (1998).

[32] Andersen, P. N., Hjarsen, T. & Williams, N. M. *Monitoring and management of high Andean biodiversity – a study from Cochabamba*, *Bolivia*. (1999).

[33] Marcora PI, Renison D, País-Bosch AI, Cabido MR, Tecco PA (2013). The effect of altitude and grazing on seedling establishment of woody species in central Argentina. Forest Ecol. Manag..

[34] Gareca EE, Martinez YY, Bustamante RO, Aguirre LF, Siles MM (2007). Regeneration patterns of Polylepis subtusalbida growing with the exotic trees Pinus radiata and Eucalyptus globulus at Parque Nacional Tunari, Bolivia. Plant Ecol..

[35] Bellis LM, Pidgeon AM, Alcántara C, Dardanelli S, Radeloff VC (2015). Influences of succession and erosion on bird communities in a South American highland wooded landscape. Forest Ecol. Manag..

[36] Fjeldså, J. & Kessler, M. *Conserving the biological diversity of Polylepis woodlands of the highland of Peru and Bolivia*. *A contribution to sustainable natural resource management in the Andes*. (NOREDECO, 1996).

[37] Lloyd H, Marsden SJ (2008). Bird community variation across Polylepis woodland fragments and matrix habitats: implications for biodiversity conservation within a high Andean landscape. Biodivers. Conserv..

[38] Herzog SK, Soria AR, Matthysen E (2003). Seasonal variation in avian community composition in a high-Andean Polylepis (Rosaceae) forest fragment. Wilson J. Ornithol..

[39] Soesbergen AJJV, Mulligan M (2014). Modelling multiple threats to water security in the Peruvian Amazon using the WaterWorld policy support system. Earth Syst. Dyn..

[40] Mulligan, M. The human water quality footprint: agricultural, industrial, and urban impacts on the quality of available water globally and in the Andean region. *Proc. Int. Conf. Integr. Water Resour. Manag. Clim. Chang*. 11 (2009).

[41] Bruijnzeel LA, Mulligan M, Scatena FN (2011). Hydrometeorology of tropical montane cloud forests: emerging patterns. Hydrol. Process..

[42] Mulligan M (2010). The Andes basins: biophysical and developmental diversity in a climate of change. Water Int..

[43] Mulligan M (2013). WaterWorld: a self-parameterising, physically based model for application in data-poor but problem-rich environments globally. Hydrol. Res..

[44] Torres RC, Renison D, Hensen I, Suarez R, Enrico L (2008). Polylepis australis’ regeneration niche in relation to seed dispersal, site characteristics and livestock density. Forest Ecol. Manag..

[45] Giorgis, M. A., Cingolani, A. M., Teich, I. & Poca, M. Can livestock coexist with Polylepis australis forests in mountains of central Argentina? Setting thresholds for a land sharing landscape. *Forest Ecol. Manag*. **457** (2020).

[46] Muñoz MA, Faz A, Acosta JA, Martínez-Martínez S, Zornoza R (2015). Effect of South American grazing camelids on soil fertility and vegetation at the Bolivian Andean grasslands. Agric. Ecosyst. Environ..

[47] Hofstede RGM, Groenendijk JP, Coppus R, Fehse JC, Sevink J (2009). Impact of Pine Plantations on Soils and Vegetation in the Ecuadorian High Andes. Mt. Res. Dev..

[48] Cierjacks A, Rühr NK, Wesche K, Hensen I (2007). Effects of altitude and livestock on the regeneration of two tree line forming Polylepis species in Ecuador. Plant Ecol..

[49] Licata JA, Gyenge JE, Fernández ME, Schlichter TM, Bond BJ (2008). Increased water use by ponderosa pine plantations in northwestern Patagonia, Argentina compared with native forest vegetation. Forest Ecol. Manag..

[50] Burgman MA, Lindenmayer DB, Elith J (2005). Managing landscapes for conservation under uncertainty. Ecology.

[51] Mulligan M (2013). WaterWorld: a self-parameterising, physically based model for application in data-poor but problem-rich environments globally. Hydrol. Res..

[52] Fjeldså J (2007). The relationship between biodiversity and population centres: the high Andes region as an example. Biodivers. Conserv..

[53] Macek P, Macková J, de Bello F (2009). Morphological and ecophysiological traits shaping altitudinal distribution of three Polylepis treeline species in the dry tropical Andes. Acta Oecol..

[54] Célleri R, Feyen J (2009). The Hydrology of Tropical Andean Ecosystems: Importance, Knowledge Status, and Perspectives. Mt. Res. Dev..

[55] Hensen I (2002). Impacts of anthropogenic activity on the vegetation of Polylepis woodlands in the region of Cochabamba, Bolivia. Ecotropica.

[56] Fjeldså J (1993). The avifauna of the Polylepis woodlands of the Andean highlands: the efficiency of basing conservation priorities on patterns of endemism. Bird Conserv. Int..

[57] Purcell J, Brelsford A, Kessler M (2002). The World’ s Highest Forest. Am. Sci..

