# Supplementary material for: Identifying trade-offs between biodiversity conservation and ecosystem services delivery for land-use decisions
Source: Sci Rep. 2020 May 14;10:7971. doi: 10.1038/s41598-020-64668-z (PMC7224365; doi:10.1038/s41598-020-64668-z)

TITLE

Identifying trade-offs between biodiversity conservation and ecosystem services delivery for land-use decisions

AUTHORS

Constance FASTRÉ (ORCID: 0000-0002-6624-3748, constancefastre@gmail.com,)[a], Hugh P. POSSINGHAM (hugh.possingham@tnc.org)[b,c], Diederik STRUBBE (diederik.strubbe@ugent.be)[a,d,e] and Erik MATTHYSEN (erik.matthysen@uantwerpen.be)[a]

AUTHOR AFFILIATIONS

[a] Evolutionary Ecology Group, Department of Biology, University of Antwerp, Universiteitsplein 1, 2610 Wilrijk, Belgium

[b] Centre of Excellence for Environmental Decisions, School of Biological Sciences, The University of Queensland, Queensland 4072, Australia

[c] The Nature Conservancy, 4245 Fairfax Drive, Arlington, VA 22203, USA

[d] Center for Macroecology Evolution and Climate, Universitetsparken 15, University of Copenhagen, 2100, Copenhagen, Denmark

[e] Terrestrial Ecology Unit (TEREC), Department of Biology, Ghent University, K.L Ledeganckstraat 32, 9000 Gent, Belgium

Corresponding author: Constance Fastré, constancefastre@gmail.com, Evolutionary Ecology Group, Department of Biology, University of Antwerp, Universiteitsplein 1, 2610 Wilrijk, Belgium, +3232651941

ELECTRONIC SUPPLEMENTARY MATERIAL

Extended methods S1: bird occurrence data collection and habitat suitability models

Bird species occurrence data were collected in the field by two skilled observers (C.F. and Hannes Ledegen) in 17 Polylepis remnants of the Tunari National Park, from September to December 2014 and 2015 (**Fig. S1**). Observers walked randomly inside the forest patches and recorded all bird species visually and aurally. Flyovers and obvious non-target species (not specifically associated with forest habitats e.g. water-birds, grassland specialists and raptors) were excluded from censuses. We generated habitat suitability models for all 35 species with more than five occurrence records, the minimum number of points necessary to produce informative models using the MaxEnt methodology (Lessmann et al. 2014). The resulting total number of available occurrences per species ranged between 8 and 361 (average of 73). Among these 35 species, eight species are particularly associated with *Polylepis* habitats throughout their range (*Polylepis*-associated species, Table 1, Fjeldså and Krabbe 1990). Three of these species, which almost exclusively rely on the tree species itself to feed at least part of the year, are further considered to be *Polylepis*-dependent (Table 1, Fjeldså and Krabbe 1990).

Spatially explicit habitat suitability estimates were obtained using the maximum entropy method (Maxent version 3.3, Phillips & Dudık 2008) as it was shown to outperform other modeling methods (e.g. see Hernandez et al. 2008). As predictor variables, we used fourteen environmental layers covering the study area: (1-5) five climatic layers (annual mean temperature, mean diurnal range, temperature seasonality, annual precipitation and precipitation seasonality) obtained from the WorldClim database (Hijmans et al. 2005) and interpolated at a 30-meters resolution (matching the resolution of the topographical data, see below), following a methodology from Zimmermann and Roberts 2000; (6) the ombrothermic index, calculated as 10*(Pp/Tp) where Pp is the yearly positive precipitation in mm and Tp is the yearly positive temperature; a digital elevation model (ASTER-GDEM, 30 m resolution, http://gdex.cr.usgs.gov/gdex/) from which we derived four topographic variables, (7) slope, (8) aspect direction, (9) the Topographic Wetness Index (TWI, which describes

the rainwater accumulation potential) and (10) the Topographic Position Index (TPI, which indicates the relative position of each pixel compared to the position of all pixels located in a 25 meter radius around the focus pixel). Finally, we derived at 30 meters resolution from the LANDSAT satellite image mentioned above, (11) current land use cover (as explained in the section above, **Fig. S2**); (12) the Normalized Difference Vegetation Index (NDVI, a measure of greenness) and (13) the Tasseled Cap Wetness Index, which reflects the actual moisture content of the soil. Finally, we included a (14) soil type layer provided by a local GIS center (CLAS, 6 soil types). Given the large number of species modelled (35 bird species), MaxEnt models were run using default settings, relying on 1.000 pseudo-absences randomly selected across the TNP. Model predictive accuracy was overall good, as for all species, model TSS value was > 0.81 (Supplementary **Table S1**).

Extended methods S2: estimation of *Polylepis* cover per planning unit

The LANDSAT image was recorded in August 2015, the driest month of the year, when the contrast between dry puna grasses/agricultural crops and evergreen *Polylepis* (and other native) trees or exotic plantations is maximal. The image was automatically classified using the IMPACT toolbox (The satellite IMagery ProCessing Toolbox, Simonetti et al., 2015). Using ground-truthed data, we assigned each one of the 42 land cover classes generated by the IMPACT tool to one of the six main land covers recorded in the TNP (Puna grasslands/bare soil/agricultural field; *Polylepis* forests; Exotic plantations; Native shrublands; Water bodies; Bogs, **Fig. S2**). We calculated the *Polylepis* cover as the percentage of current *Polylepis* forest cover per planning unit.

Extended methods S3: description of current agricultural and grazing practices currently being carried out in the TNP (Sanabria Siles et al. 2012)

The type of agricultural activities in the TNP are mainly determined by the elevation and topographic complexity characterizing the locations where they are carried out. Most of such activities currently consist in extensive agriculture and subsistence agriculture usually carried out at high altitudes on steep slopes (>15%) and characterized by moderate to low productivity due to limited water availability. Potatoes are most commonly grown in the area, and to a lesser extent are grown other tubers (yam,

papalisa and mashua), cereals (oat, barley, wheat, maize), quinoa and legumes (broad beans, lupin and beans). At lower altitudes crops include vegetables such as onions and garlic, different types of flowers supplied to the market in the city and fruits for personal consumption such as pears and avocado. Although these areas are mainly used for agricultural purposes, they are also used to raise draft animals such as oxens, horses, donkeys or mules as well as poultry. More productive, intensive agricultural practices are solely carried out in the most fertile lower areas of the Slope with access to water supplies. The majority of the crop and husbandry production is aimed for local production although surplus and flowers produced in the area are sold in the market of Cochabamba.

Table S1 Model predictive accuracy (TSS value) and threshold habitat suitability value (threshold value) calculated using the TSS statistics for each species modeled with Maxent

Species	TSS value	Threshold value	Species	TSS value	Threshold value
Sappho sparganurus	0.94	0.16	*Troglodytes aedon*	0.86	0.21
Patagona gigas	0.95	0.14	*Spinus crassirostris*	0.84	0.29
Colaptes rupicola	0.88	0.19	*Atlapetes fulviceps*	0.87	0.29
Psilopsiagon aymara	0.92	0.13	*Zonotrichia capensis*	0.93	0.16
Melanopareia maximilianus	0.94	0.16	*Agelaoides badius*	0.94	0.15
Scytalopus simonsi	0.90	0.14	*Oreopsar bolivianus*	0.94	0.19
Cinclodes fuscus	0.87	0.41	*Myioborus brunniceps*	0.96	0.07
Cinclodes atacamensis	0.88	0.16	*Saltator aurantiirostris*	0.93	0.17
Sylviorthorhynchus yanacensis	0.92	0.10	*Compsospiza garleppi*	0.96	0.18
Lepasthenura fuliginiceps	0.93	0.13	*Poospizopsis hypocondria*	0.90	0.24
Phacellodomus striaticeps	0.90	0.18	*Conirostrum binghami*	0.94	0.12
Asthenes dorbygnyi	0.95	0.20	*Conirostrum cinereum*	0.96	0.27
Asthenes heterura	0.94	0.21	*Sicalis olivascens*	0.91	0.16
Ampelion rubrocristatus	0.95	0.06	*Phrygilus atriceps*	0.94	0.15
Anairetes flavirostris	0.81	0.34	*Catamenia inornata*	0.89	0.18
Anairetes parulus	0.95	0.17	*Diglossa carbonaria*	0.94	0.20
Ochthoeca oenanthoides	0.92	0.18	*Pseudosaltator rufiventris*	0.94	0.18
Ochthoeca leucophrys	0.92	0.13			

Fig. S1 Map of the Tunari National Park (Bolivia), showing elevation (in meters) and Polylepis forest fragments surveyed for the presence of bird species. Forest patches belonging to the same watershed are shown inside the frames. Names of the watersheds are indicated in bold outside the frames, in black for *Polylepis subtusalbida* and in grey for *Polylepis lanata* tree species.

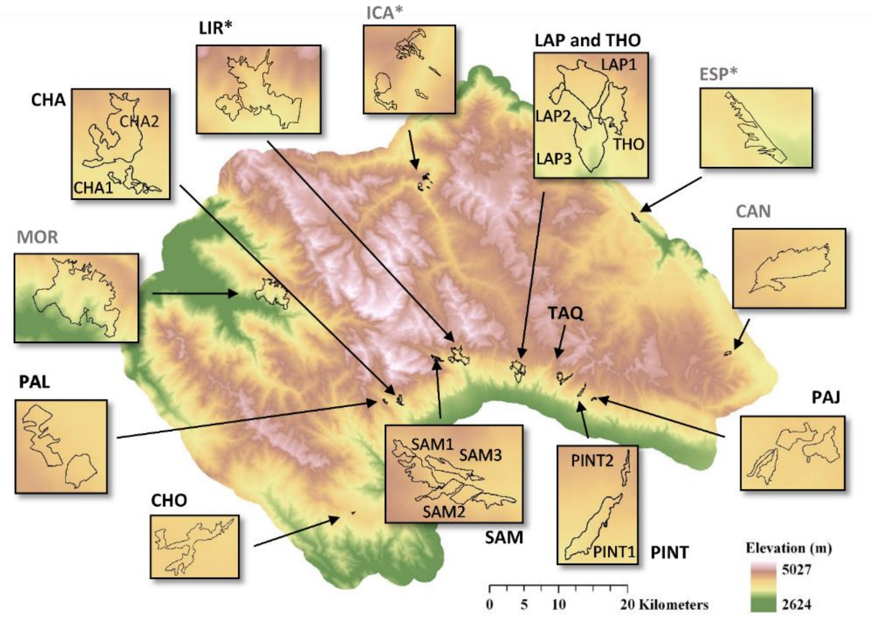

Fig. S2 Proportion of study area (%) allocated per zone (AGR = Agricultre, GR = Grazing, FOR = Forestry, AFO = Agroforestry and CON = Conservation) of the zoning plans for the six MarZone scenarios

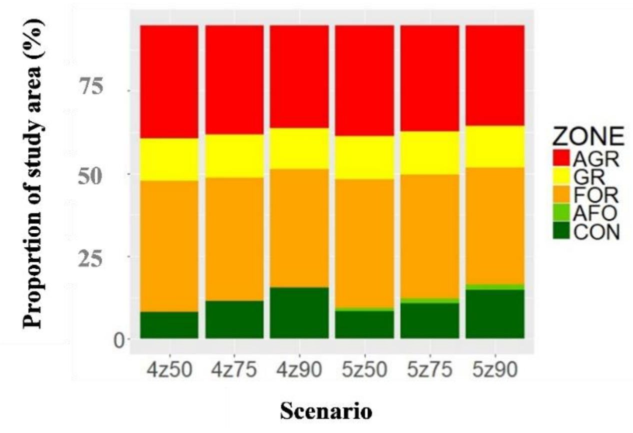

Fig. S3 Total opportunity costs (USD) of the six zoning plans generated by MarZone

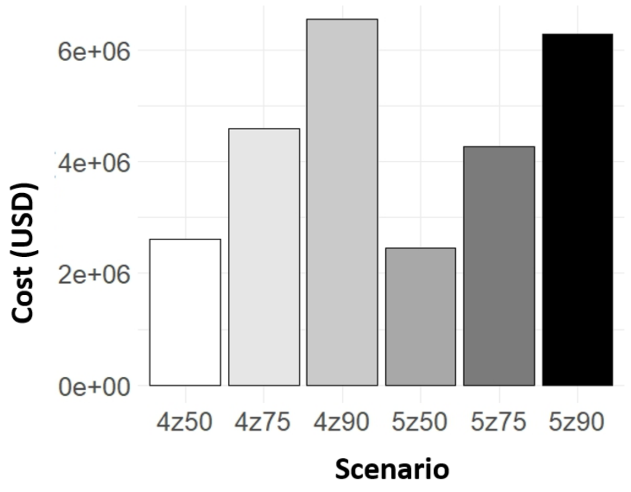

Fig. S4 Land cover map extracted from the Landsat-8 satellite image (courtesy of the U.S. Geological Survey) using the IMPACT toolbox (https://forobs.jrc.ec.europa.eu/products/software/impact.php)

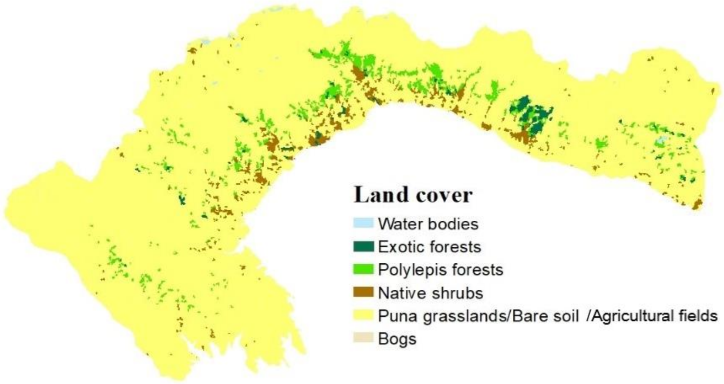

Fig. S5 Best land-use potential in the study area

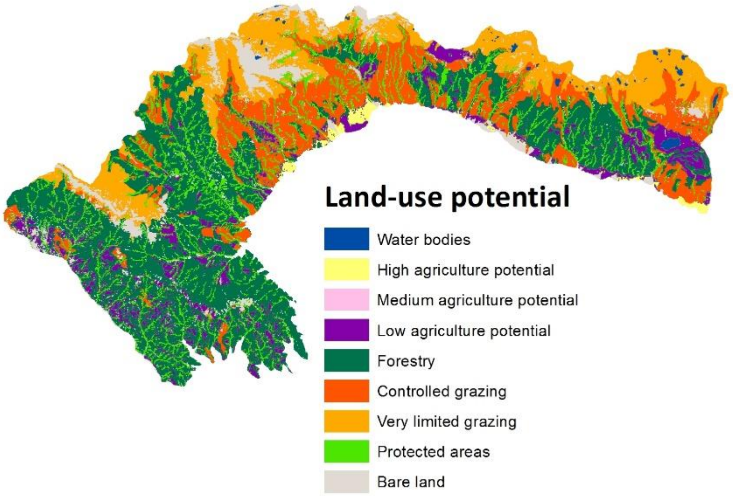

Fig. S6 Map of the log10 of the current annual erosion levels (mm) on the Southern Slope of the TNP modeled with AguAAndes

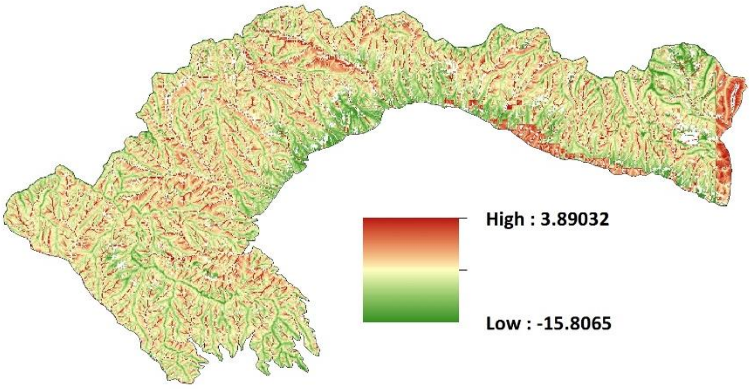

Fig. S7 Map of the log10 of the current annual runoff levels (m^3) on the Southern Slope of the TNP modeled with AguAAndes

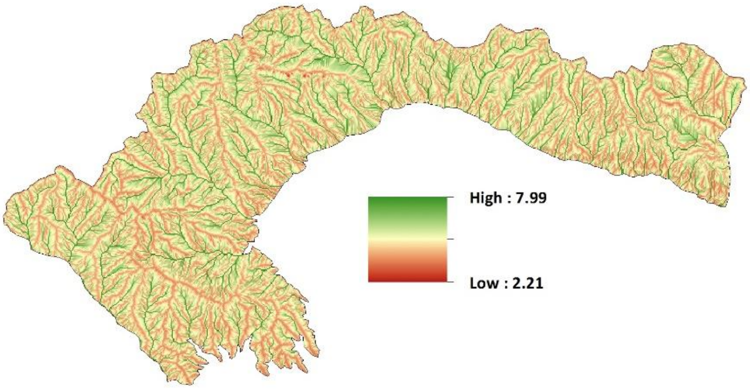

Fig. S8 Map of the current annual water stress levels (%) on the Southern Slope of the TNP modeled with AguAAndes

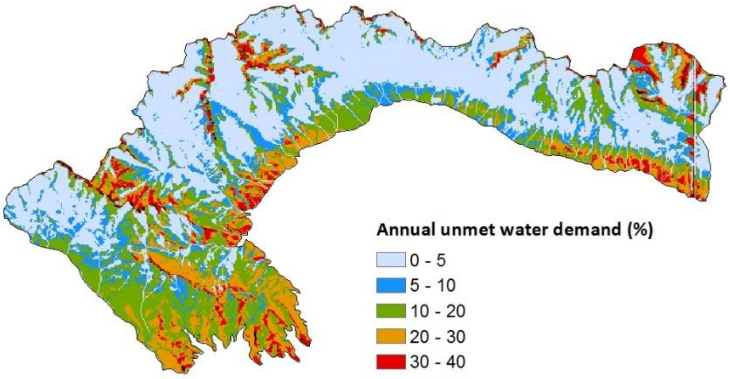

Fig. S9 Map of the current water pollution levels (%) on the Southern Slope of the TNP modeled with AguAAndes

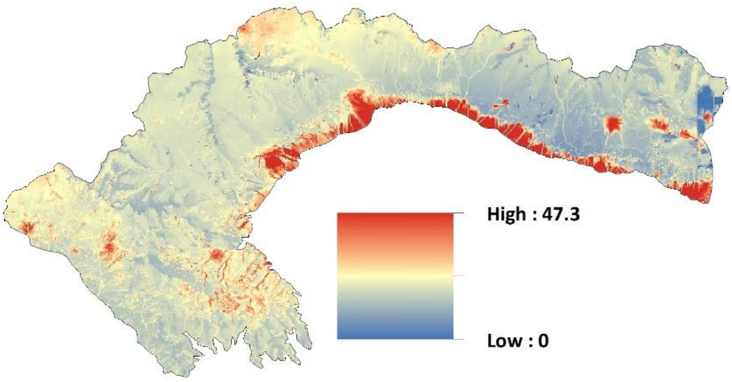

Supplement: Supplementary file 1 — Supplementary information. [file 41598_2020_64668_MOESM1_ESM.pdf]
